# Application of the amplification-free SERS-based CRISPR/Cas12a platform in the identification of SARS-CoV-2 from clinical samples

**DOI:** 10.1186/s12951-021-01021-0

**Published:** 2021-09-08

**Authors:** Jiajie Liang, Peijun Teng, Wei Xiao, Guanbo He, Qifang Song, Ying Zhang, Bin Peng, Gan Li, Liangshan Hu, Donglin Cao, Yong Tang

**Affiliations:** 1 Department of Bioengineering, Guangdong Province Engineering Research Center of Antibody Drug and Immunoassay, College of Life Science and Technology, Jinan University, Guangzhou, 510632 People’s Republic of China; 2 Department of Laboratory Medicine, Guangdong Second Provincial General Hospital, Guangzhou, 510317 People’s Republic of China; 3 Guangdong Biowings Tech Limited, Foshan, 528000 People’s Republic of China

**Keywords:** Sliver nanoparticle, Surface-enhanced Raman scattering, COVID-19, SARS-CoV-2, CRISPR, Cas12a, Biosensors

## Abstract

The control of contagious or refractory diseases requires early, rapid diagnostic assays that are simple, fast, and easy-to-use. Here, easy-to-implement CRISPR/Cas12a-based diagnostic platform through Raman transducer generated by Raman enhancement effect, term as SERS-CRISPR (S-CRISPR), are described. The S-CRISPR uses high-activity noble metallic nanoscopic materials to increase the sensitivity in the detection of nucleic acids, without amplification. This amplification-free platform, which can be performed within 30–40 min of incubation time, is then used for detection of SARS-CoV-2 derived nucleic acids in RNA extracts obtained from nasopharyngeal swab specimens (n  =  112). Compared with the quantitative reverse transcription polymerase chain reaction (RT-qPCR), the sensitivity and specificity of S-CRISPR reaches 87.50% and 100%, respectively. In general, the S-CRISPR can rapidly identify the RNA of SARS-CoV-2 RNA without amplification and is a potential strategy for nucleic acid point of care test (POCT).

**Supplementary Information:**

The online version contains supplementary material available at 10.1186/s12951-021-01021-0.

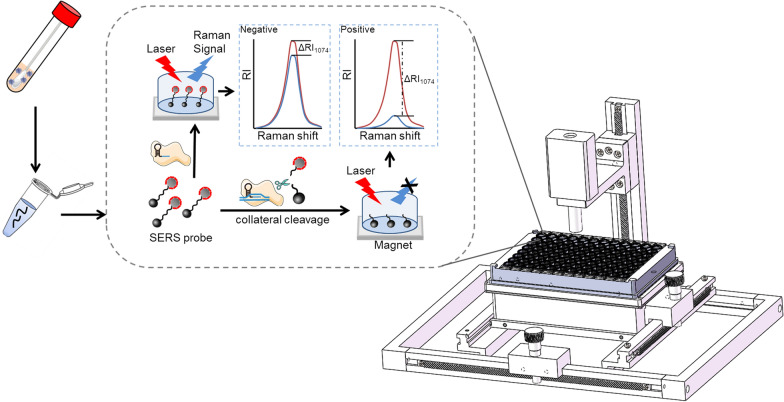

## Introduction

Contagious diseases including severe acute respiratory syndrome and refractory diseases such as prostatic cancer endanger public health and property [1]. In early January 2020, the coronavirus disease 2019 (COVID-19), a cluster of cases with pneumonia, due to infection with the severe acute respiratory syndrome coronavirus 2 (SARS-CoV-2), was first reported [2]. Soon afterward, this new coronavirus paralyzed the world because of the long incubation periods of patients and the absence of obvious biomarkers [3, 4]. Therefore, early, rapid, and easy-to-use diagnostic method is essential to identify patients and implement appropriate containment strategies, thereby preventing spread of the virus [5–7].

The RNA-guide endonuclease named clustered regularly interspaced short palindromic repeats (CRISPR)-associated (Cas) enzyme, has been exploited as an efficient genome editing tool [8, 9]. In addition to targeting endonuclease activity (targeted cleavage), CRISPR types III, V, and VI RNA-guided nucleases (Cas12, Cas13, and Cas14) display collateral target-activated, nonspecific single-stranded nucleic acid hydrolysis activity (collateral cleavage), which supports the current CRISPR/Cas-based nucleic acid diagnostic assays with advantages of reduced reaction time and amplification of self-signal [10–12]. Because of their specificity and the relatively simple design of guide RNAs, it is promising to utilize CRISPR/Cas enzymes nucleic acids detection through fluorescent transduction systems or lateral-flow strips [13–15]. In combination with amplification of nucleic acid, it is suggested that CRISPR/Cas-based nucleic acid diagnostic approaches can be used to test various substances, including single nucleotide polymorphisms, miRNA, methyladenosine and pathogens [16–20]. Importantly, COVID-19, as a fulminant infectious disease, can also be detected using CRISPR/Cas-based nucleic acid diagnostic methods [5, 21–23]. Although the fluorescent signal is relatively sensitive, nucleic acid amplification remains a necessary step to improve sensitivity, resulting in complex experimental operation and primer design, and thus limiting their applications [24–26].

Clinically, the lower the detection limit, the easier the patient is to identify. Therefore, it is importance of low limit of detection for clinical SARS-CoV-2 early screening. For improving the sensitive, presently, electrochemical and gas- volumetric sensing strategies have been applied in CRISPR/Cas-based biosensors, which have shown good performance and achieved amplification-free nucleic acid detection [27–30]. Noble metallic nanoscopic surfaces generate Raman enhancement effect, termed as surface-enhanced Raman scattering (SERS), an alternative high sensitive sensing strategy, also has potential application in CRISPR/Cas-based biosensors. This has resulted in the advancement of the use of SERS-based sensors [31, 32].

This study applied CRISPR/Cas12a-based methods in the development of SERS-based CRISPR diagnostic platform (S-CRISPR) because of their relative high-sensitivity of the transduction systems compared with the fluorescent transducer. Further, we used S-CRISPR to detect nucleic acids without amplification, particularly in the detection of SARS-CoV-2 derived RNA from nasopharyngeal swab samples. The S-CRISPR achieves 87.50% sensitivity and 100% specificity compared with quantitative reverse transcription polymerase chain reaction (RT-qPCR). Our results show that the amplification-free S-CRISPR has the potential for use as an early rapid screening diagnostic tool.

## Materials and methods

### Materials and reagents

The silver nitrate (AgNO_3_, 99.8%) was purchased from Sinoreagent (Shanghai, China). Tween-20 and Chloroauric acid (HAuCl_4_) were obtained from Amresco (USA). Trisodium citrate and 4-aminothiophenol (4-ATP) were obtained from Sigma-Aldrich. Magnetic beads (MBs)@Streptavidin was purchased from BEAVER (Suzhou, China). The RNase Inhibitor and the NEBuffer 2.1 were obtained from the New England Biolabs (USA). Microwell plate was obtained from CORNING, USA.

### Clinical samples collection and ethics statement

The procedures used in this study for collection and treatment of clinical samples of COVID-19 (Characteristics of patients listed in Additional file 1: Table S1) were approved by the Scientific Research Ethics Review Committee of the Guangdong Second Provincial General Hospital (Ethical Approval No. 20200915-01-01-YXKXYJ-CRB) and were in accordance with the standard operation of WHO. Patients or their legal representatives had provided informed consent. Nasopharyngeal swab samples from patients with suspected SARS-CoV-2 infection were analysed at the Department of Laboratory Medicine, Guangdong Second Provincial General Hospital. RNA was extracted from the samples using the MAGPURE RNA Kit (Hybribio, Guangdong, China) according to the manufacturer’s instructions. RNA extracts were initially used for molecular diagnosis of SARS-CoV-2 by RT-qPCR targeting N and orf1a genes using 2019-nCoV RT-qPCR Kit (DAAN Gene). Excess RNA extracts from these samples were used for results validation of S-CRISPR. Samples were randomized and validation assays were performed in a blinded manner.

### Nucleic acid preparation

The N gene fragment from the SARS-CoV-2 (Wuhan-1 strain, GenBank: MN908947) was generated by Sangon Biotech (Shanghai, China) and was incorporated into the pUC57 vector. The Certificate of Analysis (COA) report from manufacturer indicated the concentration of plasmid was identified by Ultraviolet Absorption Spectrometry (A260) and the purity was identified by A260/A280. The CRISPR-RNA (crRNA) of target sites from the genome of SARS-CoV-2 was designed by CHOPCHOP and Benchling. All other DNA and RNA used in this study were generated by the GenScript (Nanjing, China) and these sequences are listed in Additional file 1: Table S2. All RNAs were aliquoted and kept at − 80 °C for future experiments.

### Verification of the Cas12a-based assay using fluorescent

LbCas12a trans-cleavage assays were conducted as previous paper [28]. Target DNA was incubated with 50 nM LbCas12a, 62.5 nM crRNA, 40 U RNase Inhibitor and 50 nM linear ssDNA labeled with a quencher (ssDNA-FQ) and a fluorophore in 1  ×  NEBuffer 2.1 for 20 min at 37 °C and then distributed in 384-well plates for the florescent readout. To detect fluorescence, signal with excitation at 485 nm and emission at 535 nm was detected using the SYNERGY microplate reader (BioTek Instruments, H1).

For fluorescent Cas12a-based SARS-CoV-2 N gene plasmid detection, the plasmid was incubated with 50 nM LbCas12a, 62.5 nM crRNA, 40 U RNase Inhibitor and 50 nM ssDNA-FQ in 1  ×  NEBuffer 2.1 for 20 min at 37 °C and then distributed in 384-well plates for the florescent readout. SYNERGY microplate reader (BioTek Instruments, H1) with the excitation at 485 nm and emission at 535 nm was used for fluorescence detection.

### The construction of the portable Raman plate reader

The Raman plate reader was assembled using laser-cut acrylic housing and readily available consumer components. The laser module was fixed on the top of the device with up and down adjustment allowing accurate focusing. The microwell plate was fixed to a magnetic plate holder, which can be inserted in the device to measure the Raman signal.

### The procedure of SERS-based CRISPR diagnostic platform

To obtain SERS probe, we first prepared silver nanoparticle (AgNPs) according to those previously described [31]. Ultrapure water (500 mL) was used to dissolve AgNO_3_ (90 mg) and the solution was boiled. To the boiling silver nitrate solution, 1% trisodium citrate aqueous solution (10 mL) was added dropwise and the solution was vigorously stirred. The mixed solution was boiled for additional 30 min and until a stable green-gray AgNPs is produced. The AgNPs solution (10 mL) was mixed with 10 μL of 1 mM 4-ATP was consistently stirred for 1 h at room temperature. The mixture was centrifuged at 6000 rpm at 4 °C for 10 min. The resulting pellet (AgNPs@4ATP) was suspended in 10 mL ultrapure water. Then, 100 μL MBs@streptavidin were washed three times by magnetic shelf, and resuspended by 500 μL Buffer 1. 30 μL 4 mM SH-ssDNA-biotin was added to the MBs@streptavidin and incubated 30 min. The MBs-ssDNA-SH were reacted with AgNPs@4ATP for 30 min to form SERS probe. The SERS probe was washed three times and was stored at 4 °C till used.

The gene target of SARS-CoV-2 RNA was reverse transcribed by ReverTra Ace^®^qPCR RT Kit (TOYOBO, FSQ-101) following instructions of the manufacturer. The reverse transcribed product of SARS-CoV-2 RNA gene target was incubated with 50 nM LbCas12a, 62.5 nM crRNA, 40 U RNase Inhibitor and SERS probe in 1  ×  NEBuffer 2.1 at 37 °C for 20 min. The MBs were washed three times by magnetic shelf and resuspended by 100 μL ultrapure water. The well plates placed in portable Raman plate reader and the Raman signal was measured one by one using a portable Raman spectrometer (QSPEC, SmartRaman) with 300 mW power of excitation laser at 785 nm and accumulation time of 10 s. The characteristic Raman peak (1074 cm^−1^) of 4-ATP was noticeably identified and was used for analysis.

### S-CRISPR for the detection of SARS-CoV-2 from clinical samples

RNA extracts from nasopharyngeal swabs were reverse transcribe by ReverTra Ace^®^qPCR RT Kit (TOYOBO, FSQ-101) following instructions of the manufacturer. The reverse transcribed products were detected by S-CRISPR respectively according to the procedure of S-CRISPR.

### Statistical analysis

Data were analysed with the GraphPad Prism 8. All results were presented as mean  ±  standard error unless stated otherwise. The Clopper-Pearson method was used to calculate two-sided confidence intervals of specificity, sensitivity, positive predictive agreement (PPA) and negative predictive agreement (NPA).

## Results and discussions

### Verification of the Cas12a-based assay for SARS-CoV-2 RNA detection

CRISPR-Cas12a proteins are RNA-guided enzymes that can bind and cleave double-stranded DNA (dsDNA) or single-stranded DNA (ssDNA) using a RuvC catalytic pocket [33, 34]. Once the Cas12a-crRNA duplex recognizes the target DNA, Cas12a can activate its single-stranded deoxyribonuclease activity, in addition to its endonuclease activity, to nonspecifically cleave nearby ssDNAs, which is termed as trans-cleavage [35]. This high-efficiency trans-cleavage activity (approximately 1250 turnovers per second) and the crRNA-dependent sequence specificity of Cas12a have been used in the identification of nucleic acids with high specificity, sensitivity and rapidity [11]. Almost all of these assays utilized activated Cas12a to cleave the ssDNA-FQ which can then release a fluorescence signal (Additional file 1: Figure S1a).

Here, we first validated the collateral cleavage activity of Cas12a-crRNA duplex. The ssDNA-FQ was cleaved by Cas12a in response to the verified fragments of dsDNA triggers (Target DNA) confirmed the performance of the cognate crRNA (Additional file 1: Figure S1b). Cas12a with cognate crRNA detected its Target DNA at the lowest concentration of 0.01 nM (Additional file 1: Figure S2a–c) according to fluorescence readout and optimized reaction conditions (the concentration of crRNA was 62.5 nM and Cas12a was 50 nM) were selected for subsequent experiments (Additional file 1: Figure S2d). The time-course curves indicated that reaction acceleration in the first 20 min and complete reaction after 20 min, Because the point-in-time of 20 min was the smallest point-in-time which the fluorescence ratio of adjacent points-in-time that nearest 1 (< 0.99). Comparing the different rates of each point-in-time, 20 min was chosen as reaction time of trans-cleavage.

Following this, we designed crRNA for the rapid and specific detection of SARS-CoV-2 (accession NC_045512) in the N gene (Additional file 1: Figure S3a) following the World Health Organization (WHO) guidelines (January 17, 2020). Using plasmids encoding the N gene sequence of SARS-CoV-2, SARS-CoV (Accession NC_004718) and bat SARS-like CoV (Accession MG772933), we showed that the CRISPR-Cas12-based detection assay could distinguish SARS-CoV-2 from the other related coronavirus strains with no cross-reactivity (Additional file 1: Figure S3b, c). Thus, we next explored the ability of SERS-based CRISPR diagnostic platform to identify amplification-free SARS-CoV-2 RNA.

### Establishment of SERS-based CRISPR diagnostic platform for SARS-CoV-2 RNA detection

**Fig. 1:**
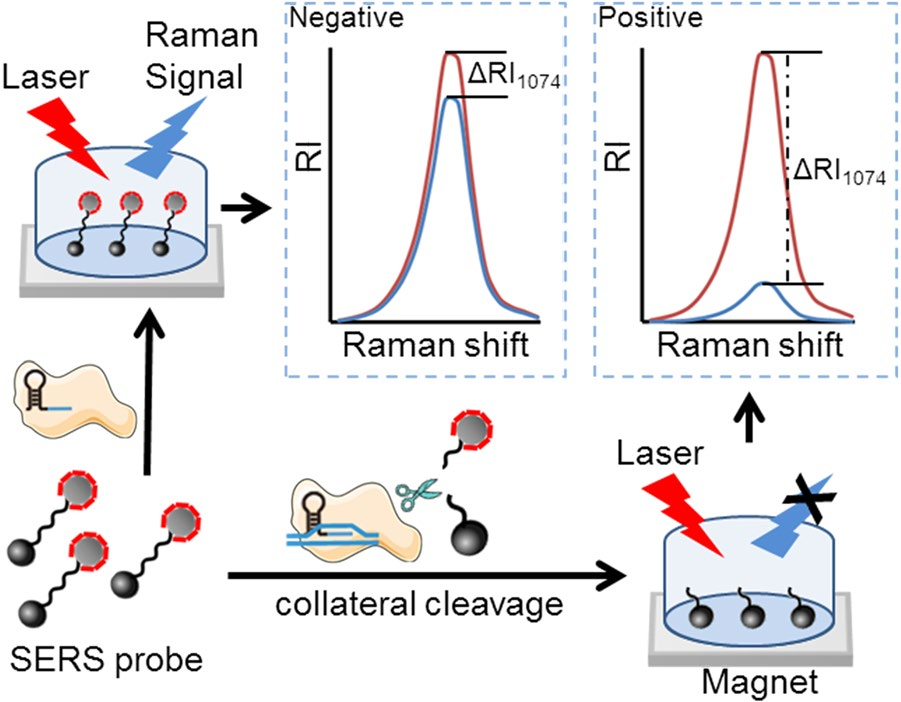
Schematic of the S-CRISPR assay

This study utilized SERS transduction for CRISPR/Cas12a-based method to identify SARS-CoV-2 RNA. Based on the findings of previous studies [31], AgNP@4ATP was used as a Raman tag and linked to MB through an ssDNA form a SERS probe. The target DNA activates the Cas12a collateral cleavage, thus removing the Raman tags off the MBs and decreasing the Raman intensity. The Raman intensity of negative quality control test is represented by red line and sample test is represented by blue line (ΔRI  =  RI_control test_ − RI_sample test_) (Fig. 1). We first prepared the SERS probe linked to AgNPs@4ATP and MBs@streptavidin through SH-ssDNA-biotin (Additional file 1: Figure S4a) and identified the concentrations of AgNP@4ATP and SH-ssDNA-biotin (Additional file 1: Figure S4b). Subsequently, the Target DNA was employed to verify that the SERS probe can be cleaved by Cas12a to change the SERS intensity (Additional file 1: Figure S4c). Importantly, the lowest detection limit was 1 fM, indicating the potential of the S-CRISPR assay to detect SARS-CoV-2 RNA.

**Fig. 2:**
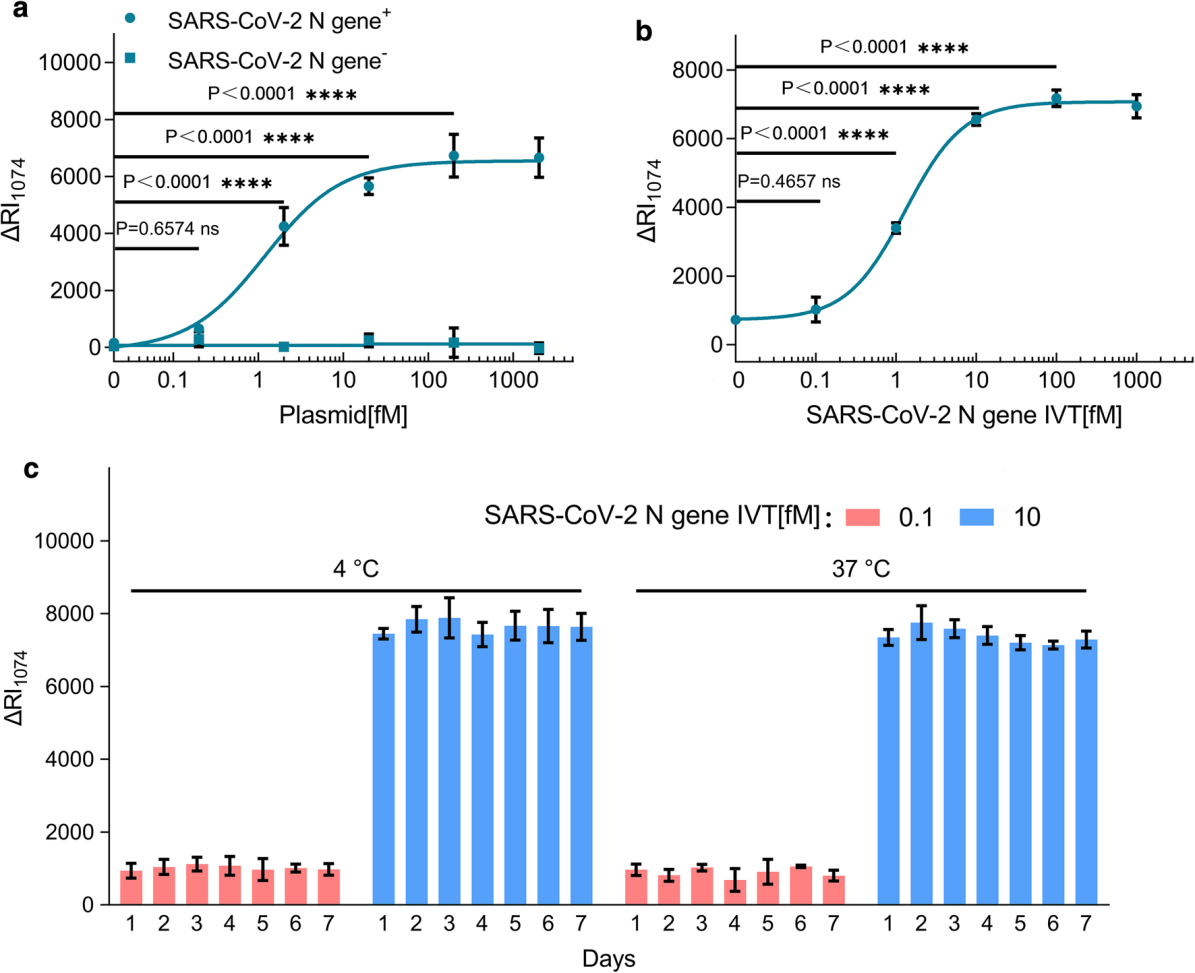
S-CRISPR for SARS-CoV-2 detection. **a** Titration of SARS-CoV-2 N gene-positive and -negative plasmids as detected by the S-CRISPR. The fitting model: [Inhibitor] vs. response—Variable slope (four parameters). Equation: Y = 6548 + (− 111.1–6548)/(1 + (1.204/X)^−0.9674^), R^2^  =  0.9665. **b** S-CRISPR was assessed using IVT RNA products from SARS-CoV-2. The fitting model: [Inhibitor] vs. response–Variable slope (four parameters). Equation: Y = 7074 + (724.2–7074)/(1 + (1.304/X)^−1.201^), R^2^  =  0.9937. **c** Initial validation of the storage life of the S-CRISPR assay. The components of the S-CRISPR assay were stored in 4 °C and 37 °C for up to 7 days, and the IVT RNA products (0.1 fM, 10 fM) were used to assess the signal change during the indicated storage period. The LoD was defined by a significant variation of Δ Raman signal at the lowest concentration. Standard deviations were determined based on three independent experiments

Next, we focused on developing the S-CRISPR for the SARS-CoV-2 detection. The SARS-CoV-2 N gene-positive plasmid was used for the verification of S-CRISPR. A curve for performance was constructed using six dilutions of an N gene-positive and -negative plasmid, with three replicates at each dilution (Fig. 2a). We confirmed the ability of the assay to generate a significant variation of ΔRaman signal at the lowest concentration of N gene-positive plasmid, and the lowest detection limit was 2 fM, indicating the feasibility of the S-CRISPR assay to detect the cDNA reverse transcribed from SARS-CoV-2 RNA. Subsequently, a synthetic, in vitro-transcribed (IVT) SARS-CoV-2 RNA gene target was used to mimic the detection of RNA viruses. A standard curve for quantitation was constructed using six dilutions of a control IVT viral nucleoprotein RNA (Fig. 2b). The analytic limit of detection (LoD) defined by a significant variation of ΔRaman signal at the lowest concentration was determined to be 1 fM. Additionally, the initial validation of the stability of the SERS probe and the Cas12a-crRNA duplex indicated no noticeable changes in the performance of the S-CRISPR assay to detect the RNA of SARS-CoV-2 up to day 7 at room temperature and 37 °C (Fig. 2c). Therefore, these findings suggest that the proposed S-CRISPR can detect SARS-CoV-2.

### Development of portable Raman plate reader for SERS-based CRISPR diagnostic platform

**Fig. 3:**
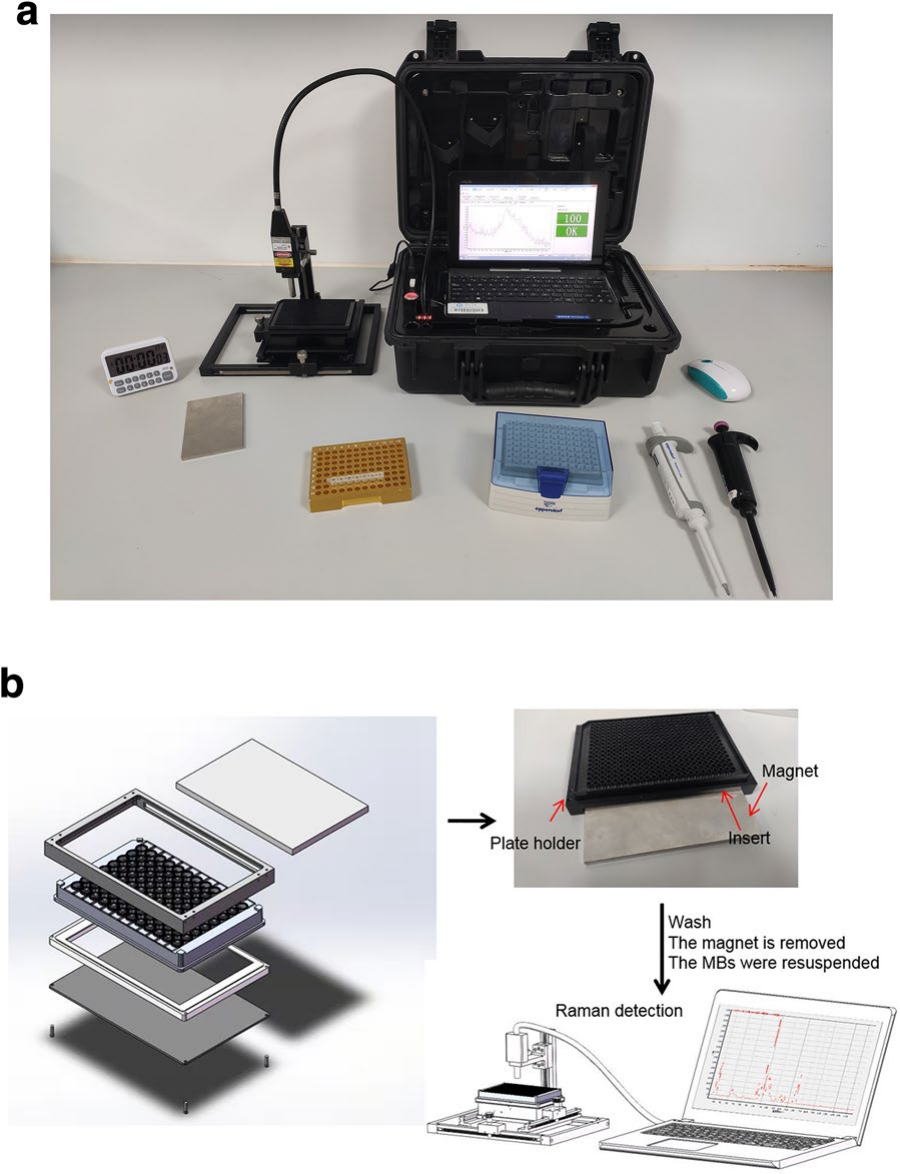
Portable Raman plate reader. **a** Schematic diagram of the low-cost, easy-to-use portable Raman plate reader developed to read the Raman output from a portable Raman spectrometer. **b** operation procedure of the portable Raman plate reader

The implementation of S-CRISPR generally requires large Raman spectrometers which may increase the assay expense and reduce the portability for application in clinical diagnosis. Currently, although portable Raman spectrometers are becoming commercially available, they lack accuracy and stability because of the large phase difference and low spatial resolution and integration (Additional file 1: Figure S5a, b). Here, we designed a portable Raman plate reader to provide robust and quantitative measurements of Raman signals with low cost (Fig. 3a). The Raman plate reader has a total cost of approximately ¥200. Once the cleavage was completed in the microwell, the custom magnet was inserted in the plate holder and the free Raman tags were washed off. After washing, the custom magnet was removed from the plate holder, and the MBs were resuspended. At this point, the plate holder can be placed into the device, and the position of the laser module can be adjusted (Fig. 3b). As a proof of concept, we evaluated the DNase cleaving activity of the SERS probe and observed significant reads (P  <  0.01) from the different concentrations of DNase with 10 replicates per concentration (Additional file 1: Figure S5c). The result indicated the stability of the portable Raman plate reader is suitable for the proposed S-CRISPR for the identification of SARS-CoV-2 from clinical samples.

### The application of SERS-based CRISPR diagnostic platform in the detection of SARS-CoV-2 from clinical samples

**Fig. 4:**
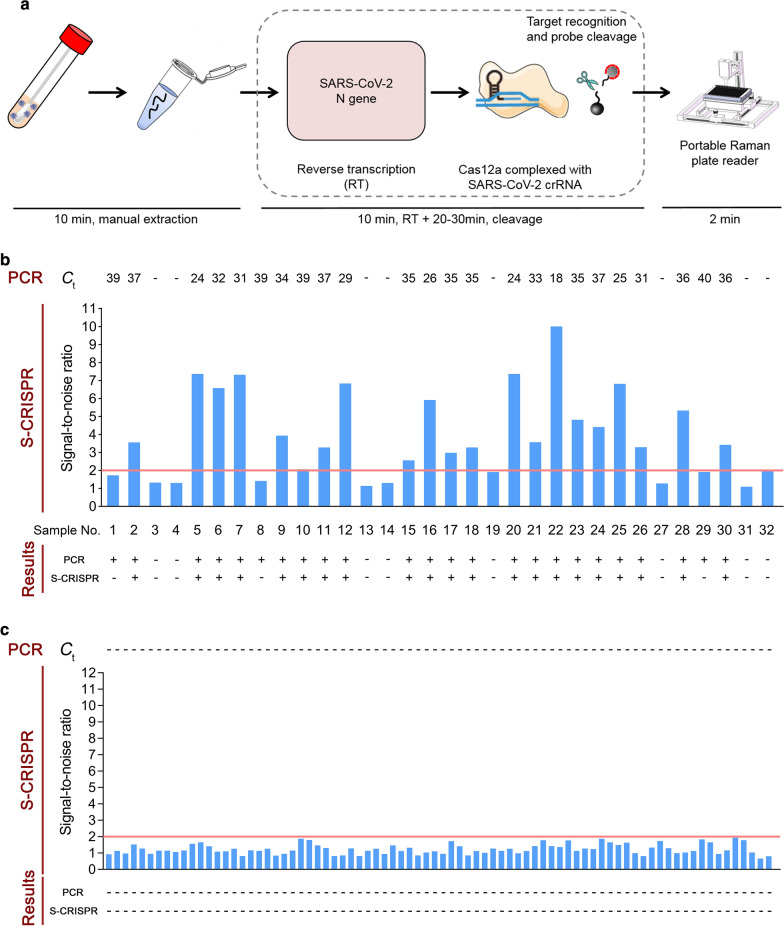
S-CRISPR for the identification of SARS-CoV-2 from nasopharyngeal swab samples. **a** Schematic of the workflow for the SARS-CoV-2 S-CRISPR. The traditional RNA extraction protocol can be used as an initial step in CRISPR-based assays and is measured using portable Raman plate reader. **b** S-CRISPR for the identification of SARS-CoV-2 in 32 clinically diagnosed COVID-19 positive samples. The 80 negative samples are shown in **c** For the ΔRI readouts, the noise is defined as the signal intensity of a negative sample (water) measured in parallel for a positive result, the threshold (pink line) of signal-to-noise ratio (S/N) is set at 2

Lastly, we validated the feasibility of the S-CRISPR for SARS-CoV-2 detection from clinical samples. The S-CRISPR workflows were validated using 112 nasopharyngeal swab samples collected from patients in the Guangdong Second Provincial General Hospital (Fig. 4a). Of the 112 patients, 32 were COVID-19-positive based on clinical diagnosis and 80 were negative (patient characteristics are listed in Additional file 1: Table S1). Because RT-qPCR was performed before the proposed CRISPR-based assays for all samples, we kept the RNA samples at − 80 °C to reduce sample degradation and performed the CRISPR-based validation experiments in the Guangdong Second Provincial General Hospital to prevent sample degradation during transportation. Among the 112 nasopharyngeal swab samples, 21 samples were identified as positive (Fig. 4b) and 91 samples were identified as negative (Fig. 4c) by N gene S-CRISPR. Compared with RT-qPCR (Table 1), the S-CRISPR was 87.5% sensitive and 100% specific. These corresponded to 100% positive predictive agreement and 96.7% negative predictive agreement for the S-CRISPR (Table 2). Compared with that of other amplification-based CRISPR assays, our proposed assay was rapid (30–40 min) and easy-to-use, which are desirable properties for early rapid screening. In addition, our S-CRISPR used similar sample collection procedures and RNA extraction methods as that in RT-qPCR, which are easy to operate. The short time spent in the development and validation of the assays, i.e.,  <  2 weeks, during the detection of SARS-CoV-2 shows that these assays can offer rapid diagnosis of infections due to emerging viruses.

**Table 1:** Concordance between the results of the RT-qPCR and S-CRISPR for the SARS-CoV-2 N gene detection

	Samples diagnosed using RT-qPCR		
	Positive	Negative	Total
S-CRISPR			
Positive	21	0	21
Negative	3	88	91
Total	24	88	112

**Table 2:** Results for the sensitivity, specificity, and predictive agreement characterizations^a^ of the S-CRISPR for the identification of the SARS-CoV-2 N gene

	Sensitivity [95% CI]	Specificity [95% CI]	PPA [95% CI]	NPA [95% CI]
S-CRISPR	87.50% (66.54–96.72)	100% (94.79–100)	100% (80.76–100)	96.70% (89.99–99.15)

^a^Values were calculated from 112 samples for clinical validation and compared with those of RT-qPCR

*CI* confidence interval

## Conclusions

The global COVID-19 pandemic highlights the need for rapid, economical, and easy-to-use diagnostic kits, which would contribute to early decisions on disease-control strategies and management of patient treatments. In this study, utilizing the high-sensitivity of SERS, we developed amplification-free SERS-based CRISPR diagnostic platform for SARS-CoV-2 detection which could save the time of nucleic acid amplification. In addition, to advance the S-CRISPR to be used in diagnostics, we designed a portable Raman plate reader to measure Raman signals with advantages of high accessibility and low-cost. The S-CRISPR was able to detect SARS-CoV-2 genes from nasopharyngeal swab samples, in the absence of amplification, thereby serving as alternatives to conventional assays.

Overall, we validated the potential of S-CRISPR for nucleic acid detection. The S-CRISPR should be further developed to detect other genes of the SARS-CoV-2 genome and thus increase their sensitivity. The automation of the portable Raman plate reader and the stability of S-CRISPR should also be explored by future studies.

## Supplementary Information

**Additional file 1: Figure S1. **Verification of the collateral cleavage activity of Cas12a using fluorescent assay. (a) Schematic of the Cas12a-based fluorescent assay; (b) The collateral cleavage of the Cas12a-crRNA duplex activated by dsDNA triggers. Randomly designed TargetDNAs activate respectiveCas12a-crRNA duplex. **Figure S2. **Confirmation of the Target DNA homologous crRNA using fluorescent assay and the optimized conditions. (a) Cas12a crRNA 1 was programmed to specifically Target DNA 1, and its time-course detection. The numbers represent the fluorescence ratio of adjacent points-in-time;(b) Cas12a crRNA 2 was programmed to specifically Target DNA 2;(c) Cas12a crRNA 3 was programmed to specifically Target DNA 3;(d) The concentrations of crRNAs and Cas12a were optimized. All the error bars are determined from three independent experiments. **Figure S3. **Confirmation of SARS-CoV-2 homologous crRNA. (a) Genome map of the SARS-CoV-2 showing crRNA. Visualization of the crRNA to identify N gene region in the SARS-CoV-2 genome; (b) crRNA specificity. Cas12a crRNA is programmed to specifically target SARS-CoV-2. The N gene crRNA used in the assay was specific for SARS-CoV-2 and failed to detect SARS-CoV and bat SARS-like coronavirus; (c) Time-course detection of the plasmids (2 nM) containing the N gene sequence of SARS-CoV-2, SARS-CoV and bat SARS-like coronavirus. The numbers represent the fluorescence ratio of adjacent points-in-time. All the error bars are determined from three independent experiments. **Figure S4. **Confirmation of the S-CRISPR assay. (a) SEM images (ZEISS ULTRA 55 field emission scanning electron microscopy) of AgNPs (І; scale bar: 200 nm; 30 K ×), SERS probe (MBs-ssDNA-AgNPs; ІІ; scale bar: 500 nm; 20 K ×), and SERS probe cleaved by Cas12a (ІІІ; scale bar: 500 nm; 20 K ×). AgNPs were successfully linked on the surfaces of the MBs and unlinked from them after cleavage; (b) The concentrations of SH-ssDNA-biotin and AgNPs@4ATP are optimized; (c) S-CRISPR assay was confirmed using Target DNA 1. All the error bars are determined from three independent experiments. **Figure S5. **Portable Raman plate reader. (a) The portable Raman spectrometer (QSPEC, SmartRaman); (b) Manualoperation procedureof the portable Raman spectrometer; (c) DNase-cleaving SERS probe and signals as detected by the portable Raman plate reader. All the error bars are determined from ten independent experiments. **Table S1. **Characteristics of clinical samples from COVID-19-suspected patients. **Table S2. **Nucleic acids used in this study.

## Data Availability

All data generated or analyzed during this study are included in the article and additional file.
