# Supplementary material for: Application of the amplification-free SERS-based CRISPR/Cas12a platform in the identification of SARS-CoV-2 from clinical samples
Source: J Nanobiotechnology. 2021 Sep 8;19:273. doi: 10.1186/s12951-021-01021-0 (PMC8424404; doi:10.1186/s12951-021-01021-0)
Supplement: Supplementary file 1 — Additional file 1: Figure S1. Verification of the collateral cleavage activity of Cas12a using fluorescent assay. (a) Schematic of the Cas12a-based fluorescent assay; (b) The collateral cleavage of the Cas12a-crRNA duplex activated by dsDNA triggers. Randomly designed TargetDNAs activate respectiveCas12a-crRNA duplex. Figure S2. Confirmation of the Target DNA homologous crRNA using fluorescent assay and the optimized conditions. (a) Cas12a crRNA 1 was programmed to specifically Target DNA 1, and its time-course detection. The numbers represent the fluorescence ratio of adjacent points-in-time;(b) Cas12a crRNA 2 was programmed to specifically Target DNA 2;(c) Cas12a crRNA 3 was programmed to specifically Target DNA 3;(d) The concentrations of crRNAs and Cas12a were optimized. All the error bars are determined from three independent experiments. Figure S3. Confirmation of SARS-CoV-2 homologous crRNA. (a) Genome map of the SARS-CoV-2 showing crRNA. Visualization of the crRNA to identify N gene region in the SARS-CoV-2 genome; (b) crRNA specificity. Cas12a crRNA is programmed to specifically target SARS-CoV-2. The N gene crRNA used in the assay was specific for SARS-CoV-2 and failed to detect SARS-CoV and bat SARS-like coronavirus; (c) Time-course detection of the plasmids (2 nM) containing the N gene sequence of SARS-CoV-2, SARS-CoV and bat SARS-like coronavirus. The numbers represent the fluorescence ratio of adjacent points-in-time. All the error bars are determined from three independent experiments. Figure S4. Confirmation of the S-CRISPR assay. (a) SEM images (ZEISS ULTRA 55 field emission scanning electron microscopy) of AgNPs (І; scale bar: 200 nm; 30 K ×), SERS probe (MBs-ssDNA-AgNPs; ІІ; scale bar: 500 nm; 20 K ×), and SERS probe cleaved by Cas12a (ІІІ; scale bar: 500 nm; 20 K ×). AgNPs were successfully linked on the surfaces of the MBs and unlinked from them after cleavage; (b) The concentrations of SH-ssDNA-biotin and AgNPs@4ATP are optimized; (c) S-CRISPR [file 12951_2021_1021_MOESM1_ESM.pdf]

Application of the Amplification-free SERS-based CRISPR/Cas12a Platform in the Identification of SARS-CoV-2 from Clinical Samples

Jiajie Liang[1, 2†], Peijun Teng[1†], Wei Xiao[3†], Guanbo He[2], Qifang Song[1], Ying Zhang[1], Bin Peng[1], Gan Li[1], Liangshan Hu[3]*, Donglin Cao[3]* and Yong Tang[1]*

[1] Guangdong Province Engineering Research Center of Antibody Drug and Immunoassay, Department of Bioengineering, College of Life Science and Technology, Jinan University, Guangzhou 510632, China

[2] Guangdong Biowings Tech Limited, Foshan 528000, China

[3] Department of Laboratory Medicine, Guangdong Second Provincial General Hospital, Guangzhou 510317, China

* Corresponding author: Liangshan Hu, Donglin Cao and Yong Tang, E-mail: liangshan8027@163.com (Liangshan Hu), caodl@126.com (Donglin Cao), tyjaq7926@163.com (Yong Tang).

[†] These authors contributed equally to this work.

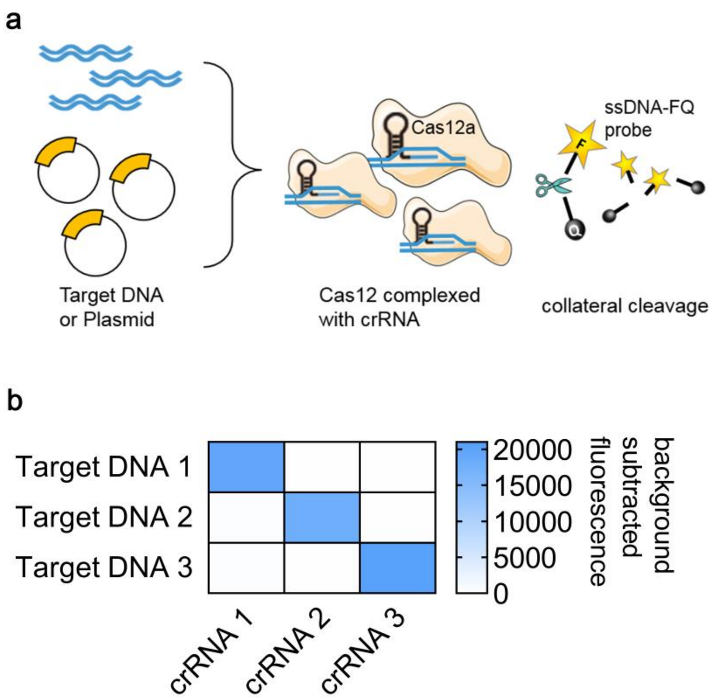

Figure S1 Verification of the collateral cleavage activity of Cas12a using fluorescent assay. (a) Schematic of the Cas12a-based fluorescent assay; (b) The collateral cleavage of the Cas12a-crRNA duplex activated by dsDNA triggers. Randomly designed Target DNAs activate respective Cas12a-crRNA duplex.

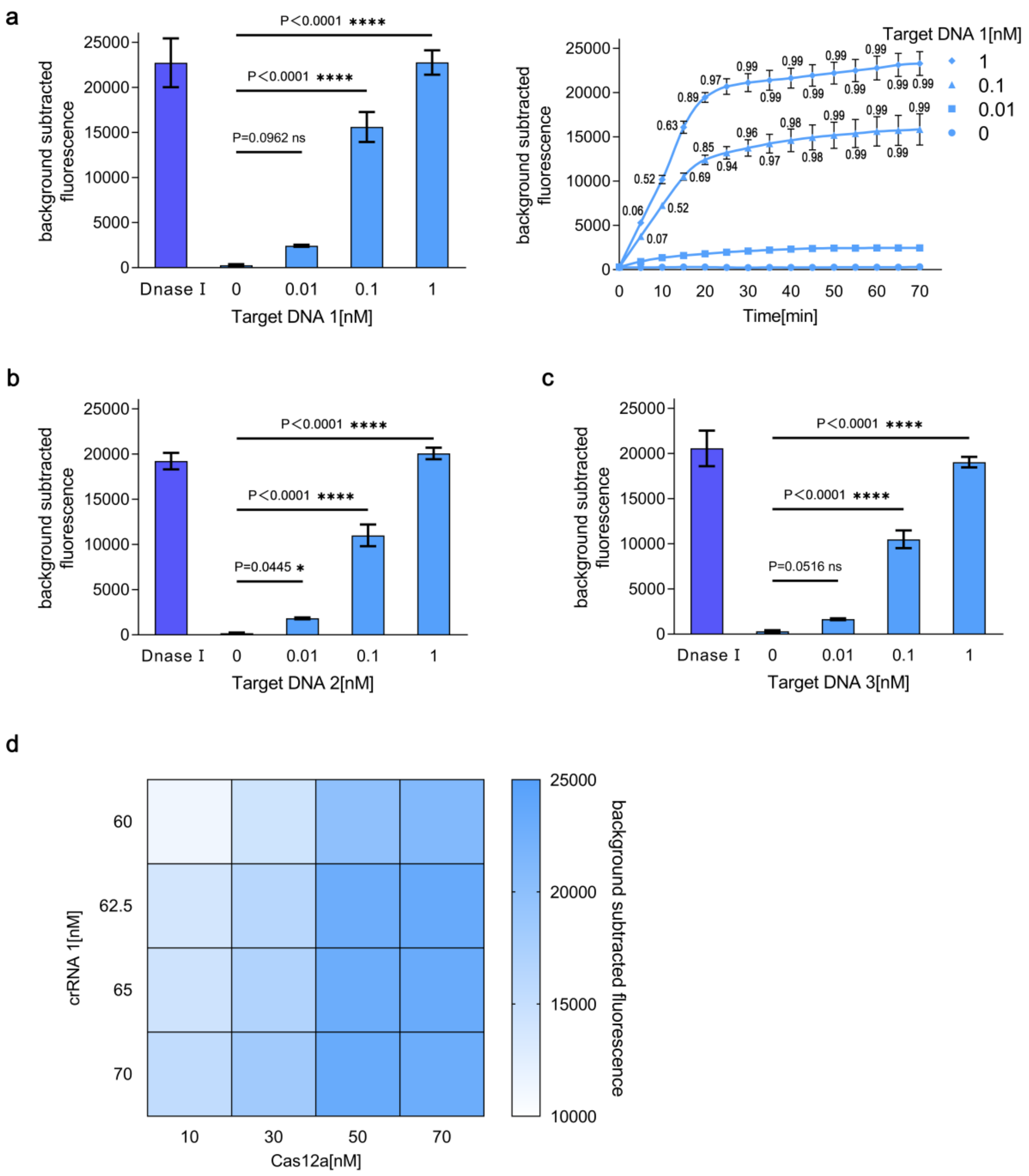

Figure S2 Confirmation of the Target DNA homologous crRNA using fluorescent assay and the optimized conditions. (a) Cas12a crRNA 1 was programmed to specifically Target DNA 1, and its time-course detection. The numbers represent the fluorescence ratio of adjacent points-in-time; (b) Cas12a crRNA 2 was programmed to specifically Target DNA 2; (c) Cas12a crRNA 3 was programmed to specifically Target DNA 3; (d) The concentrations of crRNAs and Cas12a were optimized. All the error bars are determined from three independent experiments.

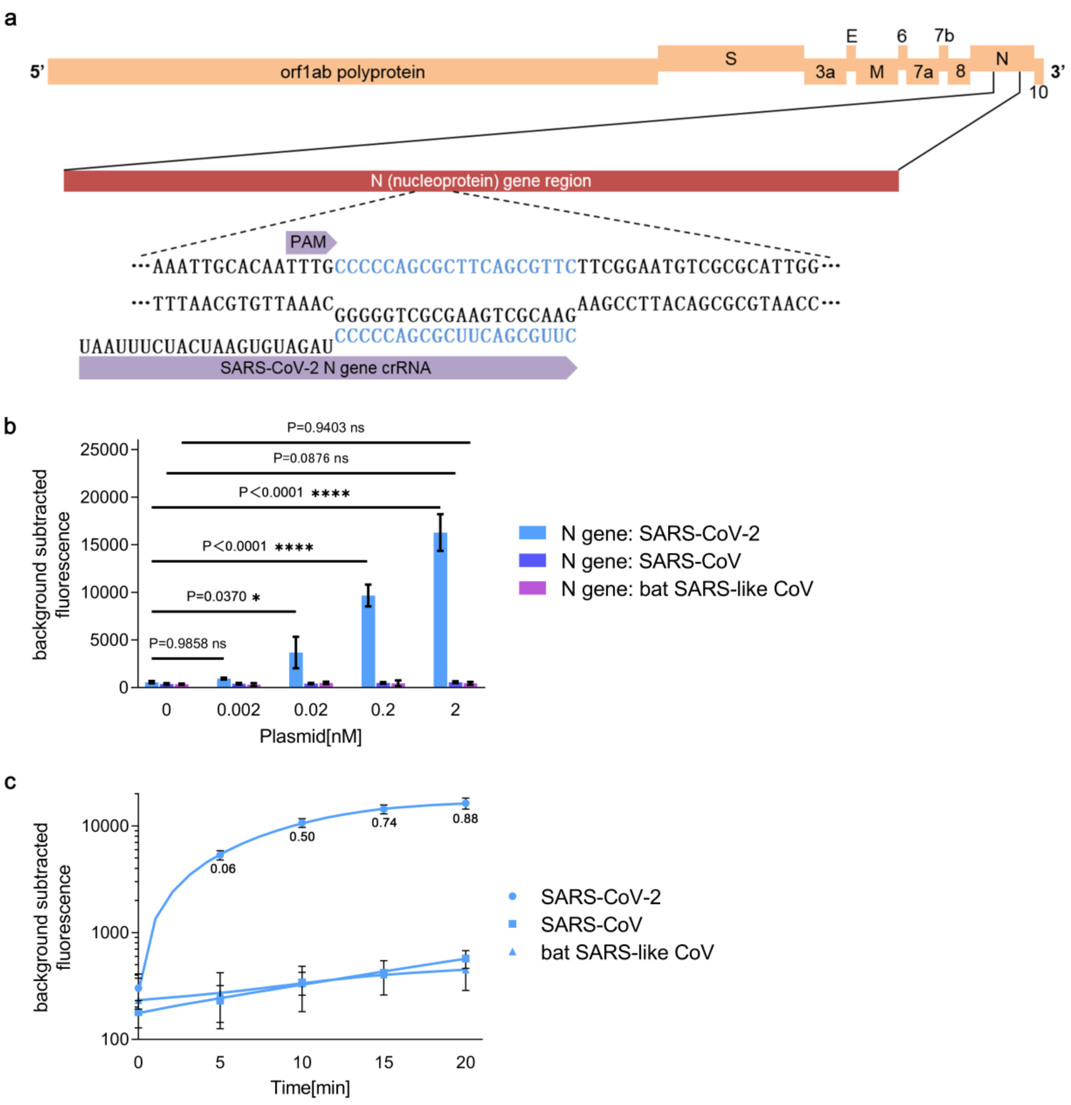

Figure S3 Confirmation of SARS-CoV-2 homologous crRNA. (a) Genome map of the SARS-CoV-2 showing crRNA. Visualization of the crRNA to identify N gene region in the SARS-CoV-2 genome; (b) crRNA specificity. Cas12a crRNA is programmed to specifically target SARS-CoV-2. The N gene crRNA used in the assay was specific for SARS-CoV-2 and failed to detect SARS-CoV and bat SARS-like coronavirus; (c) Time-course detection of the plasmids (2 nM) containing the N gene sequence of SARS-CoV-2, SARS-CoV and bat SARS-like coronavirus. The numbers represent the fluorescence ratio of adjacent points-in-time. All the error bars are determined from three independent experiments.

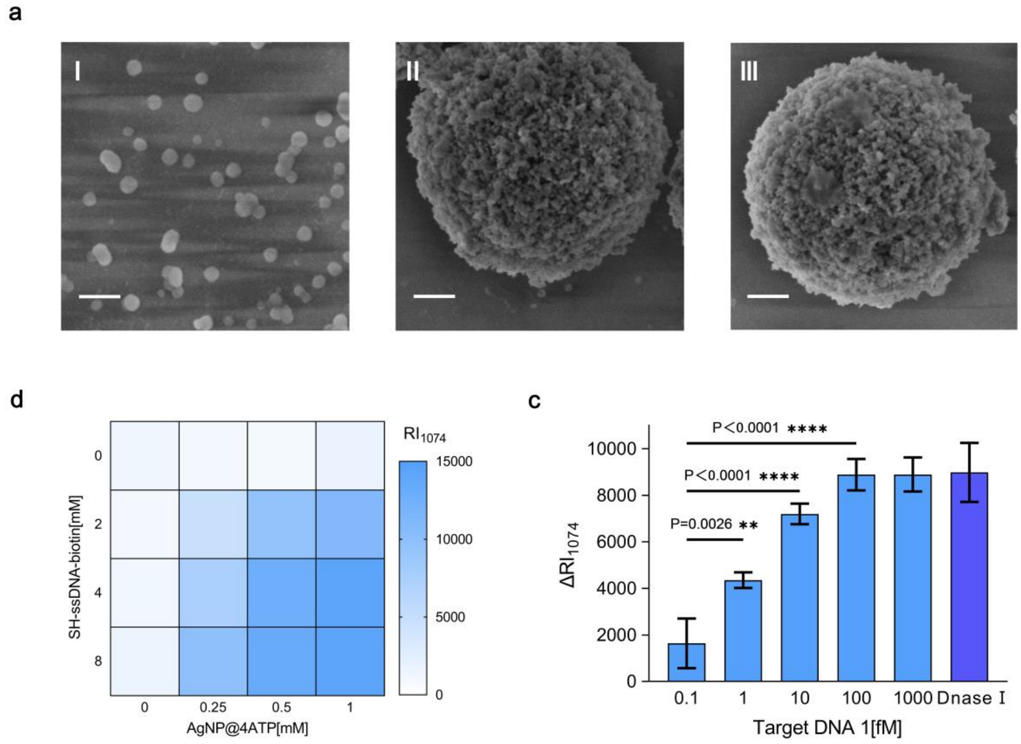

Figure S4 Confirmation of the S-CRISPR assay. (a) SEM images (ZEISS ULTRA 55 field emission scanning electron microscopy) of AgNPs (I; scale bar: 200 nm; 30 K ×), SERS probe (MBs-ssDNA-AgNPs; II; scale bar: 500 nm; 20 K ×), and SERS probe cleaved by Cas12a (III; scale bar: 500 nm; 20 K ×). AgNPs were successfully linked on the surfaces of the MBs and unlinked from them after cleavage; (b) The concentrations of SH-ssDNA-biotin and AgNPs@4ATP are optimized; (c) S-CRISPR assay was confirmed using Target DNA 1. All the error bars are determined from three independent experiments.

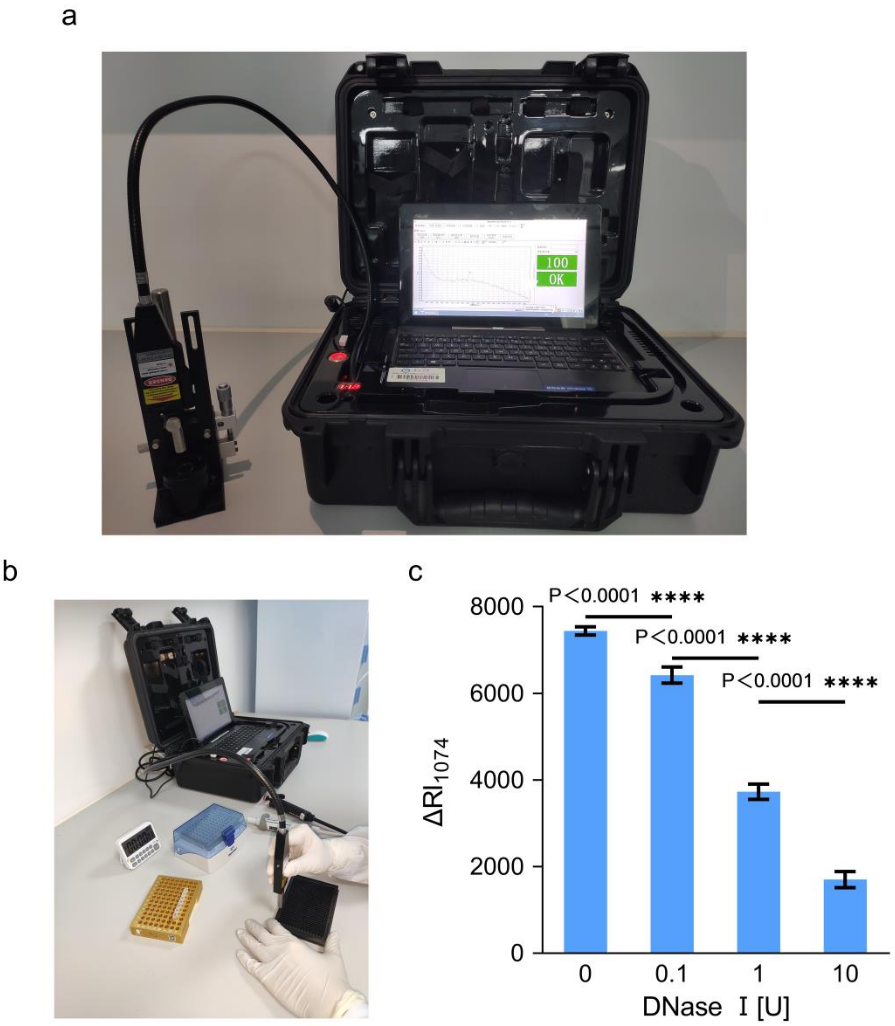

Figure S5 Portable Raman plate reader. (a) The portable Raman spectrometer (QSPEC, SmartRaman); (b) Manual operation procedure of the portable Raman spectrometer; (c) DNase-cleaving SERS probe and signals as detected by the portable Raman plate reader. All the error bars are determined from ten independent experiments.

Table S1. Characteristics of clinical samples from COVID-19-suspected patients.

Sample No.	Sex	Age	C_t of N gene PCR	C_t of orf1a gene PCR	Clinical diagnosis
1	M	67	39.35	-	Positive
2	F	56	36.73	-	Positive
3	F	38	-	-	Positive
4	M	12	-	-	Positive
5	F	48	24.48	26.88	Positive
6	M	26	31.92	33.58	Positive
7	M	58	30.91	31.64	Positive
8	F	57	39.35	40.40	Positive
9	M	43	33.66	35.49	Positive
10	M	74	38.83	41.48	Positive
11	F	65	37.37	40.83	Positive
12	M	54	28.60	31.41	Positive
13	F	36	-	-	Positive
14	M	3	-	-	Positive
15	M	53	35.33	35.47	Positive
16	M	32	25.52	26.64	Positive
17	M	32	35.47	36.37	Positive
18	F	21	34.78	38.12	Positive
19	F	55	-	-	Positive
20	M	29	24.47	25.69	Positive
21	F	31	33.23	35.51	Positive
22	M	57	17.99	19.58	Positive
23	F	54	34.94	36.37	Positive
24	M	43	36.58	37.44	Positive
25	M	31	24.96	26.48	Positive
26	F	41	30.76	32.63	Positive
27	M	25	-	-	Positive
28	M	25	35.79	36.92	Positive
29	F	37	39.42	-	Positive
30	M	61	36.44	37.42	Positive
31	F	49	-	-	Positive
32	M	67	-	40.35	Positive
33	F	21	-	-	Negative
34	F	55	-	-	Negative
35	M	24	-	-	Negative
36	M	22	-	-	Negative
37	M	32	-	-	Negative
38	F	54	-	-	Negative
39	F	55	-	-	Negative
40	F	54	-	-	Negative
41	F	55	-	-	Negative
42	M	12	-	-	Negative

43	F	55	-	-	Negative
44	F	48	-	-	Negative
45	F	49	-	-	Negative
46	M	31	-	-	Negative
47	M	32	-	-	Negative
48	F	55	-	-	Negative
49	F	56	-	-	Negative
50	F	56	-	-	Negative
51	M	43	-	-	Negative
52	F	54	-	-	Negative
53	M	29	-	-	Negative
54	M	31	-	-	Negative
55	F	49	-	-	Negative
56	M	32	-	-	Negative
57	F	56	-	-	Negative
58	F	21	-	-	Negative
59	F	30	-	-	Negative
60	F	48	-	-	Negative
61	M	74	-	-	Negative
62	M	57	-	-	Negative
63	F	55	-	-	Negative
64	F	48	-	-	Negative
65	M	57	-	-	Negative
66	F	55	-	-	Negative
67	M	12	-	-	Negative
68	M	54	-	-	Negative
69	F	54	-	-	Negative
70	F	38	-	-	Negative
71	F	55	-	-	Negative
72	F	55	-	-	Negative
73	F	53	-	-	Negative
74	F	55	-	-	Negative
75	F	21	-	-	Negative
76	F	55	-	-	Negative
77	M	24	-	-	Negative
78	M	22	-	-	Negative
79	M	32	-	-	Negative
80	F	54	-	-	Negative
81	F	55	-	-	Negative
82	F	54	-	-	Negative
83	F	55	-	-	Negative
84	M	12	-	-	Negative
85	F	55	-	-	Negative
86	F	48	-	-	Negative

87	F	49	-	-	Negative
88	M	31	-	-	Negative
89	M	32	-	-	Negative
90	F	55	-	-	Negative
91	F	56	-	-	Negative
92	F	56	-	-	Negative
93	M	43	-	-	Negative
94	F	54	-	-	Negative
95	M	29	-	-	Negative
96	M	31	-	-	Negative
97	F	49	-	-	Negative
98	M	32	-	-	Negative
99	F	56	-	-	Negative
100	F	21	-	-	Negative
101	F	30	-	-	Negative
102	F	48	-	-	Negative
103	M	74	-	-	Negative
104	M	57	-	-	Negative
105	F	55	-	-	Negative
106	F	48	-	-	Negative
107	M	57	-	-	Negative
108	F	55	-	-	Negative
109	M	12	-	-	Negative
110	M	54	-	-	Negative
111	F	54	-	-	Negative
112	F	38	-	-	Negative

Table S2. Nucleic acids used in this study.

Name	Sequence
Target DNA 1	GCTTGTGGCCGTTTA**CGTCGCCGTCCAGCTCGACC**AGGATGGGCACCACCCCGGC
Target DNA 2	GACGACAAAACTTTA**GATCGTTACGCTAACTATGA**GGGCTGTCTGTGGAATGCTA
Target DNA 3	AGTTGTGTTAGTTTA**CCTGGGTGTTCCACAGCTGA**TAGTGATTGCCTTGAATAAA
crRNA 1	UAAUUUCUACUAAGUGUAGAU**CGUCGCCGUCCAGCUCGACC**
crRNA 2	UAAUUUCUACUAAGUGUAGAU**GAUCGUUACGCUAACUAUGA**
crRNA 3	UAAUUUCUACUAAGUGUAGAU**CCUGGGUGUUCCACAGCUGA**
SARS-CoV-2 N gene	·····CACAATTTG**CCCCCAGCGCTTCAGCGTTC**TTCGG ······
crRNA (SARS-CoV-2 N gene)	UAAUUUCUACUAAGUGUAGAU**CCCCCAGCGCUUCAGCGUUC**
SARS-CoV N gene	·····CACAATTTG**CTCCAAGTGCCTCTGCATTC**TTTGG ······
bat SARS-like CoV N gene	·····CACAATTTG**CTCCAAGTGCCTCTGCATTC**TTTGG ······
Plasmid vector	pUC57
ssDNA-FQ reporter	6FAM-TTATTTATTATT-BHQ1
SERS probe	MB-streptavidin-biotin-TTTTTTTTTTTTTTTTTTTTTTTTTTTTTTTTTTTTTTTT<br>TTTTTTTTTTTTTTTTTTTTTTTTTTTTTTTTTTTTTTTTTTTTTTTTTTTTTT<br>TTTT- S-AgNP@4ATP

Note: The PAMs of Cas12a are underlined. Bold font is used to indicate the recognition region.
